# Effects of mid-gestation supplementation with dried distillers’ grains and soybean hull-based total mixed ration silages on maternal metabolism, uteroplacental hemodynamics, and offspring development in beef cows

**DOI:** 10.1093/jas/skaf430

**Published:** 2025-12-13

**Authors:** Karolina B Nascimento, Robson L Ferreira, Vinícius D Vaz, Javier A M Meneses, Daniel R Casagrande, Mateus P Gionbelli, Thiago F Bernardes

**Affiliations:** Department of Animal Science, University of Lavras, Lavras 37203-202, Brazil; Department of Animal Science, University of Lavras, Lavras 37203-202, Brazil; Department of Animal Science, University of Lavras, Lavras 37203-202, Brazil; Department of Medicine Veterinary and Animal Science, Universidad de Ciencias Aplicadas y Ambientales (UDCA), Cartagena 130001, Colombia; Department of Animal Science, University of Lavras, Lavras 37203-202, Brazil; Department of Animal Science, University of Lavras, Lavras 37203-202, Brazil; Department of Animal Science, University of Lavras, Lavras 37203-202, Brazil

**Keywords:** feed conservation, fetal programming, rumen undegradable protein, seasonal forage availability, silage-based diets

## Abstract

Maternal nutrition during gestation is a key determinant of maternal physiological adaptations and offspring developmental outcomes. This study evaluated how two total mixed ration (**TMR**)-based silages offered during mid-gestation influenced nutrient utilization, systemic physiology, uterine hemodynamics, and performance of pregnant beef cows and their offspring. Thirty-three multiparous Tabapuã cows (*Bos taurus indicus*) received one of the following treatments from day 130 to 230 of gestation: (1) TMR silage composed of Marandu grass and dried distillers’ grains plus soluble (**DDGS**; *n *= 17); or (2) TMR silage composed of Marandu grass, soybean hulls (**SH**), and urea (*n *= 16). At day 230, all cows were regrouped and managed on pasture until calving. Cow–calf pairs remained together until weaning (∼175 d), after which calves were individually housed and monitored for 100 d. Maternal digestibility, nitrogen balance, and microbial protein synthesis were assessed at d 180; uterine blood flow was evaluated by Doppler ultrasonography at day 230 and 270; and maternal blood metabolites were measured at d 230. Statistical significance was declared at *P *≤ 0.05. Cows fed DDGS-based TMR silage had greater dry matter (**DM**), crude protein, and neutral detergent fiber intakes (*P *≤ 0.01), improved DM digestibility (*P *= 0.04), and increased nitrogen retention (*P *< 0.001) compared with SH + urea-based TMR fed cows. At day 230, DDGS-fed cows exhibited greater body weight (**BW**) and body condition score (**BCS**; *P *≤ 0.04) and maintained higher BCS at calving (*P *< 0.01). Non-esterified fatty acids and beta-hydroxybutyrate concentrations were greater in SH + urea-fed cows (*P *≤ 0.02), as was preprandial urea concentration (*P *< 0.01). At day 230, cows fed SH + urea and carrying female fetuses had greater pulsatility index, resistance index, and systolic/diastolic ratio (*P *≤ 0.03). Maternal diet did not affect calf BW at birth, preweaning growth, or backgrounding performance (*P *≥ 0.20). Milk yield tended to be greater at 60 d in milk (**DIM**) in SH-fed cows (*P *= 0.07), whereas milk composition was unaffected at either 60 or 120 DIM (*P *≥ 0.21). Overall, mid-gestation supplementation with DDGS-based TMR silage improved maternal nutrient status, body condition, and uteroplacental adaptations, but no consistent effects on offspring growth were detected.

## Teaser

Feeding DDGS-based TMR silage during mid-gestation improved nutrient utilization and metabolic status in beef cows and revealed sex-specific adaptations in maternal endocrine profile and uteroplacental hemodynamics. Despite modest programming effects in the offspring, this strategy, compared with soybean hull-based TMR silage, can enhance resilience and efficiency in tropical cow–calf systems.

## Introduction

Seasonal variations in pasture availability are among the main constraints on beef cattle production in tropical regions. The pronounced asymmetry between forage abundance during the rainy season and its marked deficiency in the dry period imposes significant limitations on stocking rates and compromises the maintenance of animal productivity throughout the year (Reis et al., 2009). In beef cow–calf systems, these constraints are particularly substantial, as the dry season typically overlaps with the second and third trimesters of gestation (Santos et al., 2022). This period represents a physiologically demanding window during which fetal foundational structure of skeletal muscle tissue is established (Costa et al., 2021a; Carvalho et al., 2022). Thus, nutritional insults during this stage may compromise not only maternal metabolic status (Meneses et al., 2022, 2024), but also elicit long-term, and often irreversible, consequences on offspring performance and meat production (Costa et al., 2021b; Barcelos et al., 2022; Nascimento et al., 2022, 2024; Santos et al., 2023). In this context, strategic nutritional interventions during gestation are essential to ensure both maternal resilience and proper fetal development.

One promising strategy involves the ensiling deferred tropical grasses harvested at the end of the rainy season in combination with agro-industrial by-products to produce total mixed ration (**TMR**) silages. Given the inherently high moisture content of tropical grasses (e.g., those from the *Brachiaria* genus), combining them with by-products represents an alternative to increase dry matter (**DM**) concentration and improve fermentation quality (Bernardes et al., 2018; Gusmão et al., 2018). In Brazil, cow-calf operations are predominantly located in the Central-West region. In this area, two feed ingredients stand out: soybean hulls and dried distillers’ grains plus soluble (**DDGS**). Among farmers managing cow-calf operations, there is a culture of reducing costs in cow feeding; consequently, they have commonly used grass silage combined with soybean hulls and urea (the latter to increase protein concentration). However, previous studies have demonstrated that urea may adversely affect silage fermentation by exerting a buffering effect that impedes the normal decline in pH (Buxton and O’Kiely, 2003), while also negatively influencing feed intake (Bandla et al., 2024). Therefore, since DDGS is widely available and affordably priced in this region and also provides a high rumen undegradable protein concentration (NASEM, 2016), we hypothesized that combining grass silage with DDGS could serve as an alternative to the soybean hulls and urea combination, potentially improving silage fermentation and contributing to the metabolizable protein supply for pregnant cows. This integrative approach may represent an effective tool to mitigate the adverse effects of seasonal nutrient restrictions on maternal metabolism and fetal development, ultimately improving the biological and productive efficiency of cow–calf systems. Furthermore, within this framework, advancing scientific understanding of how the ruminal degradability profile of dietary protein influences gestational nutrition and fetal development is essential. In this sense, these differences offer a valuable opportunity to investigate and modulate dietary protein degradability with greater precision in pregnant beef cows managed under tropical conditions.

Therefore, this study aimed to evaluate the effects of two TMR silages, formulated with by-products exhibiting distinct ruminal protein degradability profiles, on nutrient intake and digestibility, maternal metabolic status, and performance outcomes in gestating beef cows and their offspring.

## Materials and Methods

### Animal welfare

All procedures were performed in accordance with ethical standards for animal experimentation and were reviewed and approved by the Institutional Animal Care and Use Committee of UFLA (CEUA/UFLA; protocol n° 010/2020). The experimental protocol complied with the ethical principles and guidelines established by the Brazilian National Council for the Control of Animal Experimentation (CONCEA), ensuring the welfare and proper handling of the animals throughout the study.

### Pre-experimental procedures

This study was performed at the Beef Cattle Research Facility of the Department of Animal Science at the Federal University of Lavras (UFLA), located in Lavras, Minas Gerais, Brazil. A completely randomized 2 × 2 factorial design was adopted, consisting of two maternal nutritional plans during gestation, based on TMR containing different silage ingredients, and fetal sex as experimental factors.

Prior to the beginning of this study, a pre-experimental phase was conducted to produce the silages used in the dietary treatments. For this purpose, an area of approximately 6.5 hectares of *Brachiaria brizantha* cv. Marandu pasture was selected based on forage uniformity, favorable topography, and the absence of physical obstructions. The pasture received maintenance fertilization with 50 kg of nitrogen per hectare, followed by a 90-d regrowth period to promote sufficient biomass accumulation.

At harvest, forage was harvested using a JF C130 pull-type forage harvester (JF Máquinas Agrícolas, Itapira, SP, Brazil) equipped with a total-area cutting platform, aiming for a uniform particle size. To ensure consistent chopping quality, the harvester blades were sharpened twice daily. The chopped forage was transported and unloaded near the storage location. A feed mixer wagon, previously tared, was loaded with chopped forage using a backhoe loader. The amount of concentrate added was proportional to the recorded forage weight. The concentrate mixtures consisted of either DDGS with a mineral supplement or soybean hulls (SH), urea, and the same mineral supplement (Bell Nutri 90; Trouw Nutrition Brasil, Campinas, SP, Brazil). The ingredients were mixed for approximately 10 minutes to ensure homogeneity and subsequently unloaded into a forage bagging machine (JF Silo Master Forage; JF Máquinas Agrícolas, Itapira, SP, Brazil). During this process, a silage additive (Fylax^®^ Forte Liquid; Selko, Trouw Nutrition Brasil, Campinas, SP, Brazil) was applied at a rate of 1 L per metric ton of fresh matter. It was diluted 1:1 in water and applied uniformly using a backpack sprayer. The resulting TMR silages were stored in silo bags (for a minimum of ∼60 d) prior to their use in the experimental period. During the formulation and combination of ingredients, two primary targets were established for both diets: approximately 30% DM to optimize silage fermentation and 9% crude protein (CP; DM basis) to meet the protein requirements of the cows.

### Experimental design, diets, and animal management

Thirty-two multiparous Tabapuã beef cows (*Bos taurus indicus*) [515 ± 59.6 kg mean body weight (**BW**), and 6 ± 0.8 body condition score] were used in this study. Estrous synchronization was conducted using a fixed-time artificial insemination protocol. Cows subjected to three insemination attempts using semen from different males; those that failed to conceive were subsequently exposed to natural mating for a 30-d period. Among the cows that conceived, 23 were successfully bred via fixed-time artificial insemination and 10 through natural service. Pregnancy diagnosis and fetal sex determination were performed via transrectal ultrasonography at approximately 60 d of gestation by a trained veterinarian.

At the beginning of mid-gestation (∼100 d), pregnant cows were removed from a pasture of *Brachiaria brizantha* cv. Marandu and housed in a tie-stall with individual pens (20 m^2^ per pen, including a 6 m^2^ roofed area), equipped with concrete floors, individual feed bunks, and water troughs. After a 30-d adaptation period, at 130 ± 4 d of gestation, one of two dietary treatments consisting of different TMR silages was randomly assigned to the cows: (1) DDGS group (*n *= 17; 8 carrying male fetuses and 9 carrying females)—TMR composed of Marandu grass and DDGS, or (2) SH+Urea group (*n *= 16; 7 carrying males and 9 carrying females)—TMR composed of Marandu grass, soybean hulls, and urea (Table 1). The mid-gestation period was selected as the supplementation window based on the theoretical timeline of skeletal muscle development proposed by Du et al. (2010), and due to its potential to improve maternal body condition, given that early and late gestation are characterized by homeorhetic adaptations to lactation and accelerated fetal growth, respectively, particularly in the context of breeding season standardization in tropical regions (Meneses et al., 2024). From 130 to 230 d of gestation, cows were fed their respective TMRs twice daily (07:00 and 16:00 h). Feed was offered ad libitum, with daily adjustments to ensure approximately 5% refusals. Clean water was available continuously. Weekly, feed offered and refusals were sampled, and composite samples were stored for subsequent chemical analysis. All samples were then ground to pass through a 1-mm screen and analyzed for nutrient composition. Dry matter, organic matter (**OM**), ether extract (**EE**), and crude protein (**CP**) contents were determined according to standard procedures (AOAC, 1990). Neutral detergent fiber corrected for ash and nitrogen (NDFcp) was determined as described by Mertens et al. (2002). Non-fiber carbohydrates (NFC) and total digestible nutrients (TDN) were estimated using the equations described by Detmann and Valadares Filho (2010) and NRC (2001), respectively.

**Table 1.** Ingredient inclusion and chemical composition of the diets (% of DM; n = 14) fed to cows during mid-gestation

Item	Feeding regimen during gestation	
	DDGS^1^	SH+Urea^2^
***Ingredient inclusion, % of DM***		
**Forage (*Brachiaria brizantha* cv. Marandu)**	86.3	86.3
**Soybean hulls**	–	11.6
**Urea**	–	1.1
**Dried distillers’ grains**	12.7	–
**Mineral mixture**	1.0	1.0
***Chemical composition, g/kg of DM***		
**Dry matter**	288 ± 30.1	282 ± 28.9
**Ash**	73 ± 4.7	68 ± 5.4
**Neutral detergent fiber**	652 ± 6.5	683 ± 26.5
**Ether extract**	20 ± 4.2	15 ± 2.1
**Crude protein**	92 ± 6.8	79 ± 7.0
**Non-fiber carbohydrates**	162 ± 6.8	156 ± 6.8
**Total digestible nutrients**	521 ± 6.8	488 ± 6.8
***Fermentation profile, g/kg of DM***		
**Lactic acid**	47 ± 14.0	21 ± 10.9
**Acetic acid**	30 ± 6.4	32 ± 6.5
**Propionic acid**	6 ± 2.2	13 ± 2.0
**Butyric acid**	4 ± 0.9	12 ± 4.6
**Ethanol**	6 ± 3.9	4 ± 1.7

1 Total mixed ration containing grass silage and dried distillers’ grains plus soluble.

2 Total mixed ration containing grass silage, soybean hulls, and urea.

At approximately 230 d of gestation, cows were transferred to a single 6.7-hectare deferred pasture of *Brachiaria brizantha* cv. Marandu, with an initial forage availability of ∼9.3 metric tons of DM per hectare. The pasture had a chemical composition of 56 ± 1.8 g/kg CP and 750 ± 8.1 g/kg NDF (DM basis; CP and NDF were analyzed as described previously). Cows were managed under a continuous stocking method during late gestation and received a low-intake protein supplement (3 g/kg of live weight) composed of 9% ground corn, 55% soybean meal, 18% urea, and 18% mineral mixture, resulting in a final supplement with 760 g/kg CP (CP was analyzed as described previously), 562 g/kg non-protein nitrogen, and 512 g/kg TDN (TDN was calculated as described previously).

Of the 33 cows initially included, three experienced calf losses during the first few days after parturition, resulting in 30 cow–calf pairs during the suckling phase. Consequently, the dataset for the lactation period comprised the following groups: DDGS-male (*n *= 7), DDGS-female (*n *= 8), SH+Urea-male (*n *= 6), and SH+Urea-female (*n *= 9). Following calving, standard neonatal procedures were implemented. Colostrum intake was ensured within the first hours of life. Newborns also received routine sanitary care, including umbilical cord disinfection and antiparasitic treatment. During the cow–calf phase, all pairs were managed as a single group on pasture. Full body weight measurements were taken at 10, 60, and 120 d of age, as well as at weaning.

Calves were weaned in a single management at an average age of 193 ± 11 d. Immediately thereafter, they were transferred to an intensive system for evaluation. A total of 29 calves (12 males and 17 females) were included in the post-weaning confined backgrounding phase, as one animal had to be removed from the study. After weaning, calves were housed in individual pens and underwent a 7-d adaptation period to acclimate to the experimental diet, housing conditions, and handling procedures. During this period, all animals were offered the same diet ad libitum (Table 2). Feed offered and orts were measured daily to estimate individual DM intake. Diet formulation and composition were established according to weekly DM determinations, actual nutrient concentrations, and corresponding feed batching records. Representative samples of each ingredient were collected weekly and preserved at −20°C until subsequent nutrient analyses. Following weekly DM assessment, ingredient samples were composited monthly to obtain representative pooled samples, which were then subjected to comprehensive chemical analyses. Orts samples collected during this period were processed and analyzed following the same procedures. Animals were fed twice daily (at 06:00 and 15:00 h), and clean water was available at all times. During the post-weaning phase, average daily gain (ADG) was evaluated at 35, 65, and 95 d of confinement after a 12-h feed and water withdrawal.

**Table 2.** Ingredient inclusion and chemical composition of the diets (% of DM) fed to young bulls and heifers during background phase

Ingredient inclusion	% of DM
**Corn silage**	71
**Dried distillers’ grains (DDGS)**	17.2
**Soybean hulls**	8.0
**Urea**	0.5
**Mineral supplement^¹^**	3.3
***Chemical composition***	***g/kg of DM***
**Dry matter**	471.2
**Crude protein**	141.6
**Neutral detergent fiber**	471.5
**Ether extract**	53.5
**Ash**	93.4
**Starch**	251.4

1 Guaranteed analysis per kilogram of mineral supplement: Ca, 132.5 g; P, 30 g; Na, 80 g; K, 50 g; Mg, 68 g; S, 25 g; Zn, 1,220 mg; Cu, 330 mg; F, 500 mg; Mn, 950 mg; Co, 20 mg; I, 24 mg; Se, 6 mg; monensin, 650 mg; vitamin A, 67,000 IU; vitamin D, 9,500 IU; vitamin E, 950 IU.

### Measurements, analysis, and calculations

#### Maternal and gestational weights

During gestation, cow BW was recorded after a 16-h withdrawal from both feed and water, and this measurement was defined as pregnant body weight (BWp). Weighing was performed in the morning, prior to feeding. Body condition score (BCS) was also assessed using a 9-point scale, where 1 represents emaciated and 9 represents obese animals (Nicholson and Butterworth, 1986). Scoring was conducted independently by three trained evaluators through visual inspection and palpation, and the final BCS was calculated as the mean score across anatomical regions evaluated by each assessor. Based on BWp and BCS, estimates of pregnant empty body weight (EBWp), non-pregnant empty body weight (EBWnp), and gestational components (PREG) were obtained using predictive equations developed by Gionbelli et al. (2015). First, EBWp was calculated from BWp, and subsequently partitioned into EBWnp and PREG. The PREG component represented the additional weight attributed to gestation and included the gravid uterus (GUdp; the difference between pregnant and non-pregnant uterus weight) and the increase in udder weight due to pregnancy. Thus, EBWp was expressed as the sum of EBWnp and PREG.

#### Maternal blood parameters

Blood samples were collected from pregnant beef cows at 230 d of gestation (end of the dietary treatment period), prior to the morning feeding. For glucose and urea analyses, additional samples were collected 4 h post-feeding. Samples (10 mL) were obtained via jugular venipuncture using vacutainer tubes (First Lab, São José dos Pinhais, PR, Brazil), immediately placed on ice, centrifuged at 2,700 rpm for 20 min, and stored at –20°C until analysis. All samples were analyzed in duplicate. Blood glucose concentrations were determined using a commercial kit (Glucose Liquiform, Labtest, Lagoa Santa, MG, Brazil) and a colorimetric method based on the glucose oxidase–peroxidase enzymatic reaction. Plasma urea–N concentrations were measured using a commercial kit (Urea CE, Labtest, Lagoa Santa, MG, Brazil) by enzymatic colorimetric end-point method. Serum concentrations of D-3-hydroxybutyrate (**BHBA**) were determined using a kinetic enzymatic method with a commercial kit (Randox Laboratories Ltd, Antrim, UK). Concentrations of non-esterified fatty acids (**NEFA**; ELK Biotechnology Co., Denver, CO, USA), insulin-like growth factor-1 (**IGF-1**; Sigma-Aldrich, St Louis, MO, USA), and insulin (Sigma-Aldrich, St Louis, MO, USA) were measured using commercial enzyme-linked immunosorbent assay kits.

#### Transrectal Doppler evaluation of uterine blood flow dynamics

Uterine artery blood flow was evaluated at the end of the supplementation period (∼230 d of gestation) and during the prepartum period (∼270 d) using a Doppler ultrasound device operating in color and spectral modes, equipped with a 7.0 MHz transrectal linear probe (UST‐5813‐5; Corometrics Medical Systems, USA). The uterine artery ipsilateral to the gravid horn was identified by transrectal palpation, and once the characteristic pulsatile flow was visualized, the Doppler gate was adjusted to obtain clear and stable waveforms. Measurements included: resistance index (**RI**), pulsatility index (**PI**), and systolic/diastolic (**S/D**) ratio were calculated as follows: RI = (peak systolic velocity − end-diastolic velocity)/peak systolic velocity; PI = (peak systolic velocity − end-diastolic velocity)/mean velocity. All Doppler parameters were automatically generated by the device’s integrated analysis software, and all scans were performed by the same trained operator to minimize interobserver variation.

#### Apparent total tract digestibility, nitrogen balance, and microbial crude protein synthesis of beef cows

Apparent total tract digestibility of DM, CP, and NDF was evaluated at 180 d of gestation during a 4-d collection period. During this period, the feed offered and orts were weighed and sampled for subsequent determination of nutrient concentrations and calculation of apparent total tract digestibility coefficients. Moreover, total fecal output was continuously collected every 24 h. Sampling was conducted by gathering feces directly from droppings on a sanitized concrete surface to prevent contamination. At the end of each 24-h collection, fecal material was weighed, thoroughly mixed, and a representative daily subsample was obtained and frozen at −20°C until further analysis. These subsamples were subsequently dried in a forced-air oven at 55°C for 72 h and ground using a 1-mm sieve to ensure homogeneity. A composite fecal sample was then constituted for each animal, considering the proportion of daily fecal output and DM concentration from each subsample. Apparent total-tract digestibility coefficients were calculated by the ratio of nutrient intake to fecal nutrient excretion throughout the collection period. Simultaneously, spot urine samples were collected twice daily in the morning and afternoon, over four consecutive days at alternating times (day 1 at 06:30 and 12:30 h; day 2 at 08:00 and 14:00 h; day 3 at 09:30 and 15:30 h; and day 4 at 11:00 and 17:00 h). Samples were used to estimate nitrogen balance and microbial crude protein (**MCP**) synthesis. Total urinary nitrogen was determined using the micro-Kjeldahl method. Urinary creatinine concentrations were measured using a commercial enzymatic kit (Creatinine K016, Bioclin, Belo Horizonte, Brazil). Allantoin concentrations were determined calorimetrically as described by Chen and Gomes (1992), and uric acid concentrations were estimated from allantoin values following the equation proposed by Santos et al. (2016). Urinary volume was indirectly estimated based on creatinine concentration (Santos et al.). The total urinary excretion of purine derivatives (allantoin + uric acid) was calculated and used to estimate absorbed purines, correcting for endogenous excretion and recovery rate as described by Prates et al. (2012). Ruminal MCP synthesis was subsequently estimated from absorbed purines using established stoichiometric relationships. Nitrogen balance was determined by quantifying nitrogen intake and urinary nitrogen excretion, allowing the evaluation of nitrogen retention and excretory losses under the experimental conditions.

#### Assays for estimating forage intake during the cow-calf phase

Two forage intake assays were conducted at 60 and 120 d of lactation to estimate the intake of DM, CP, and NDF by lactating cows. For this purpose, a combination of indigestible neutral detergent fiber (**iNDF**) (Valente et al., 2011) and titanium dioxide (**TiO_2_**) (Titgemeyer et al., 2001) was used as internal and external markers, to estimate fecal output and forage intake, respectively. Titanium dioxide was administered once daily at 0700 h in paper cartridges containing 10 g of TiO_2_ per cow via esophageal probe, for 10 consecutive days. Fecal samples were collected using the spot sampling technique on the last 4 d of marker administration, with collections performed twice daily (at 0700 and 1830 h). Representative pasture samples were collected during the trial using the hand-plucking technique (manual grazing simulation). Fecal samples collected from each animal were pooled by animal and assay, and dried in a forced-air oven at 55°C for 72 h. After drying, samples were ground through a 1- and 2-mm screens using a Wiley-type mill. Forage and fecal samples were analyzed for DM, CP, and NDF, according to the standard procedures. Indigestible NDF content in forage and feces was determined after 288 h of in situ ruminal incubation using rumen-cannulated beef cows. Fecal output was estimated by dividing the amount of TiO_2_ administered daily by its concentration in the feces. Subsequently, forage DM intake was estimated by multiplying the fecal output by the concentration of iNDF in the feces and dividing the result by the concentration of iNDF in the forage.

#### Milk production and composition

Cows were hand-milked once in the morning (0600 h) to assess milk yield and composition. Calves were separated from their dams for approximately 12 h prior to milking to avoid nursing interference. Milk ejection was pharmacologically induced by intramuscular injection of 2 mL of oxytocin (Ocitocina Forte UCB; Uzinas Chimicas Brasileiras S/A, Jaboticabal, Brazil) at 0600 h. Following milk letdown, complete udder evacuation was performed. The total milk volume was weighed, and aliquots (∼30 mL) were collected into sterile plastic vials containing a bronopol preservative tablet (D & F Control Systems Inc., San Ramon, CA, USA). Milk samples were stored at 4°C and subsequently sent to a certified laboratory specializing in dairy quality analysis for determination of individual milk composition. Daily milk yield was estimated according to the approach described by Nascimento et al. (2022).

#### Ultrasonographic assessments

Ultrasound images of the carcass were acquired by a trained technician using an Aloka 500-V ultrasound system (Corometrics Medical Systems, Wallingford, CT) equipped with a 3.5-MHz, 17.2-cm linear array transducer. Briefly, images were obtained to quantify the *Longissimus* muscle area (**LMA**), subcutaneous fat thickness (**SFT**) over the *Longissimus* muscle, rump muscle length (**RML**), and rump fat thickness. The LMA and SFT measurements were performed between the 12th and 13th ribs, capturing approximately 75% of the ventral length of the *Longissimus* muscle. The RML and rump fat thickness were assessed at the junction between the *biceps femoris* and *gluteus medius* muscles, located between the ischium and ilium and aligned parallel to the vertebral column. Image analysis was conducted using the BioSoft Toolbox II for Beef software (Biotronics Inc., Ames, IA, USA).

### Statistical procedures

A completely randomized design with a 2 × 2 factorial arrangement was used, with maternal nutrition and calf sex as fixed effects. Data were analyzed using the following baseline model:


$${\text{Y}}_{ijk}=\mu +{\text{D}}_{i}+{\text{S}}_{j}+{\left(\text{DS}\right)}_{ij}+{\text{C}}_{ijk}+\varepsilon_{ijk}$$

where **Y_*ijk*_**: observed response variable; **μ**: overall mean; **D_*i*_**: fixed effect of the *i*-th level of maternal dietary treatment (2 levels); **S_*j*_**: fixed effect of the *j*-th level of calf sex (2 levels); **(DS)_*ij*_**: interaction between maternal diet and calf sex; **C_*ijk*_**: covariates tested, including: dam’s parity, initial empty BW, initial BCS, gestation length, calf age and/or BW at the evaluation date, dam’s genetic merit index for growth traits (**GEN**); **ε_*ijk*_**: residual error, assumed to be normally distributed (ε_*ijk*_∼N(0,σ_e_^2^)).

Covariates were initially included in the model and retained when significant (*P *< 0.05). Non-significant covariates were excluded through a backward stepwise approach. The GEN index was derived from the Tabapuã Genetic Improvement Program database and was calculated based on expected progeny differences (EPDs) for birth BW and BW at weaning, 12, and 18 months of age. Model assumptions (normality and homoscedasticity of residuals) were verified through residual diagnostics. Outliers were identified based on studentized residuals and removed iteratively. Statistical significance was declared at *P *≤ 0.05, and trends were discussed when 0.05 < *P *≤ 0.10.

## Results

### Nutritional parameters of beef cows during gestation

No FR × OS interaction was detected for DM, CP, or NDF intake during mid-gestation (*P *≥ 0.79; Table 3). However, cows fed the DDGS-based diet had greater (*P *< 0.01) DM, CP, and NDF intakes than those fed the SH + urea-based diet. A FR × OS interaction was observed for CP digestibility (*P *= 0.01), where cows carrying female fetuses and fed the SH + urea-based TMR diet showed reduced CP digestibility compared with all other groups. Moreover, DM digestibility was greater (*P *= 0.04) in cows fed DDGS than in those fed SH + urea. No main effects or interactions were detected for NDF digestibility (*P *≥ 0.76; Table 3). There were no FR × OS interactions for nitrogen balance variables or MCP synthesis (*P *≥ 0.17; Table 3) during mid-gestation. Cows fed DDGS had greater (*P *< 0.001) N intake and N balance compared with those fed SH + urea. A tendency for greater fecal N output was observed in cows fed DDGS compared with SH + urea (*P *= 0.06), whereas urinary N output was not affected by any of the fixed effects (*P *≥ 0.35). Microbial CP synthesis tended to be greater in cows fed DDGS compared with those fed SH + urea (*P *= 0.07).

**Table 3.** Effects of dietary treatments during mid-gestation and offspring sex on intake, total-tract digestibility, nitrogen balance, and microbial crude protein synthesis of pregnant beef cows

Item	Feeding regimen (FR)		Offspring sex (OS)		SEM	*P*-value		
	**DDGS^1^** ** (*n *= 17)**	SH + Urea^2^ (*n *= 16)	**Female** **(*n *= 17)**	**Male** **(*n *= 16)**		FR	OS	FR × OS
***Intake, kg/day***								
**DM**	7.18	5.26	6.05	6.39	1.409	<0.01	0.51	0.93
**CP**	0.67	0.42	0.55	0.53	0.108	<0.01	0.30	0.90
**NDF**	4.88	3.91	4.48	4.31	0.903	<0.01	0.60	0.79
***Total tract digestibility, g/kg DM***								
**DM**	569	523	545	547	15.8	0.04	0.89	0.61
**CP**	676	637	640	674	16.4	0.02	0.03	0.01
**Female**	680^a^	600^b^						
**Male**	673^a^	675^a^						
**NDF**	591	596	591	597	13.1	0.82	0.76	0.95
***Nitrogen balance and microbial crude protein synthesis (MCP), g/day***								
**Nitrogen intake**	114.9	81.6	96.4	100.0	5.93	<0.001	0.65	0.84
**Urinary nitrogen output**	10.1	8.68	8.85	9.90	1.06	0.35	0.47	0.78
**Fecal nitrogen output**	38.1	33.0	35.6	35.5	1.97	0.06	0.96	0.32
**Nitrogen balance**	64.26	41.16	50.89	54.53	4.50	<0.001	0.55	0.63
**MCP**	809	594	703	700	76.4	0.07	0.98	0.17

1 Total mixed ration containing grass silage and dried distillers’ grains.

2 Total mixed ration containing grass silage, soybean hulls, and urea.

### Maternal performance during gestation

At 130 d of gestation, no significant effects of maternal dietary treatment, offspring sex, or their interaction were detected for any of the maternal performance variables evaluated (*P *≥ 0.10; Table 4), suggesting a uniform baseline across groups prior to the initiation of nutritional interventions. At 230 d of gestation, biological differences emerged as a function of maternal dietary treatment (Table 4). Cows fed a DDGS-based TMR silage exhibited greater BWp compared to those fed SH + urea-based TMR silage (*P *= 0.04). This increase was accompanied by an enhancement in EBWp (*P *= 0.04) and a tendency for greater EBWnp (*P *= 0.06) in the same group. Additionally, BCS was greater in the DDGS group (6.28 vs. 5.52; *P *= 0.01). However, no effects of any of the factors studied on PREG were observed at 230 d of gestation (*P *≥ 0.10). At parturition, no FR × OS interaction was detected for any of the maternal outcomes evaluated (*P *≥ 0.21). In addition, BWp, EBWp, and EBWnp did not differ between groups (*P *≥ 0.36; Table 4). Moreover, PREG compounds were not affected by the maternal feeding regimen adopted during mid-gestation (*P *= 0.14). On the other hand, BCS was greater in cows fed the DDGS-based diet at calving (4.56 vs. 4.10; *P *< 0.01) compared to those fed SH + urea-based TMR silage.

**Table 4.** Effects of maternal dietary treatments during mid-gestation and offspring sex on maternal performance during gestation in beef cows

Item	**Feeding regimen** ** (FR)**		**Offspring sex** **(OS)**		SEM	*P*-value		
	**DDGS^1^** **(*n *= 17)**	SH + Urea^2^ (*n *= 16)	**Female** **(*n *= 17)**	**Male** ** (*n *= 16)**		FR	OS	FR × OS
***Measurements at 130 days of gestation (initial weights)***								
**Cow BWp, kg**	524.7	538.1	521.6	541.2	11.64	0.66	0.22	0.55
**Cow EBWp, kg**	472.3	472.2	472.3	472.3	0.054	0.30	0.56	0.77
**Cow EBWnp,^3^ kg**	465.2	465.3	465.6	464.9	0.322	0.95	0.11	0.31
**Cow BCS**	5.95	5.92	6.11	5.75	0.183	0.89	0.18	0.11
**PREG,^4^ kg**	6.96	7.12	6.65	7.44	0.316	0.71	0.10	0.25
***Measurements at 230 days of gestation (end of TMR supplementation period)***								
**Cow BWp, kg**	542.8	495.9	498.9	539.7	16.30	0.04	0.08	0.28
**Cow EBWp, kg**	486.0	442.2	445.1	483.0	15.13	0.04	0.08	0.27
**Cow EBWnp,^3^ kg**	450.0	409.8	412.1	447.7	14.80	0.06	0.09	0.36
**Cow BCS**	6.28	5.52	6.03	5.77	0.203	0.01	0.35	0.85
**PREG,^4^ kg**	31.7	34.7	31.1	35.2	2.63	0.23	0.10	0.11
***Measurements at parturition***								
**Cow BWp, kg**	532.0	529.8	509.8	551.9	14.49	0.92	0.04	0.25
**Cow EBWp, kg**	494.6	497.2	477.2	514.7	15.23	0.90	0.06	0.21
**Cow EBWnp,^3^ kg**	439.0	419.7	411.9	446.9	16.25	0.36	0.11	0.51
**Cow BCS**	4.56	4.10	4.51	4.15	0.51	<0.01	0.06	0.46
**PREG,^4^ kg**	94.14	75.12	76.63	92.64	9.31	0.14	0.20	0.77

1 Total mixed ration containing grass silage and dried distillers’ grains.

2 Total mixed ration containing grass silage, soybean hulls, and urea.

3 Maternal empty body weight excluding the gravid uterus and pregnancy-related udder development.

4 Pregnancy-associated tissues, calculated as the weight of the gravid uterus minus that of the nonpregnant uterus, plus udder accretion attributable to gestation.

### Maternal blood metabolites and hormonal profile

A significant FR × OS interaction was observed for IGF-1 concentrations (*P *< 0.001; Table 5). Among cows fed the DDGS-based TMR diet, those carrying male fetuses exhibited lower IGF-1 concentrations compared with those carrying female fetuses (68.2 vs. 87.0 ng/mL). Additionally, among cows gestating male fetuses, those fed the SH + urea-based diet had greater IGF-1 concentrations than their counterparts fed DDGS-based TMR diet (85.2 vs. 68.2 ng/mL). A trend toward an FR × OS interaction was verified for insulin levels (*P *= 0.09; Table 5), where cows gestating male offspring and fed DDGS-based TMR diet tended to exhibit greater insulin concentrations than their counterparts fed SH + urea. Moreover, cows fed DDGS-based TMR diet had lower plasma NEFA concentrations (*P *= 0.02) and BHBA concentrations (*P *< 0.01) compared with those fed SH + urea-based silage. Similarly, plasma urea concentrations measured at 0 h post-feeding were lower in DDGS-fed cows (*P *< 0.01; Table 5), whereas no dietary effect was detected at 4 h post-feeding (*P *= 0.14). No effects of maternal diet were observed for plasma glucose concentrations at either timepoint (*P *≥ 0.16).

**Table 5.** Effects of maternal dietary treatment during mid-gestation and offspring sex on maternal blood metabolites, hormonal profile, and uterine hemodynamics in beef cows during gestation

Item	**Feeding regimen** **(FR)**		**Offspring sex** ** (OS)**		SEM	*P*-value		
	**DDGS^1^** ** (*n *= 17)**	SH + Urea^2^ (*n *= 16)	**Female** ** (*n *= 17)**	**Male** ** (*n *= 16)**		FR	OS	FR × OS
***Blood parameters***								
**IGF-1, Ng/mL**	77.6	79.5	80.4	76.7	2.74	0.63	0.34	<0.001
**Female**	87.0^a^	73.7^a,b^						
**Male**	68.2^b^	85.2^a^						
**Insulin, µUI/mL**	17.2	13.1	15.8	14.5	7.99	0.17	0.69	0.09
**NEFA, mmol/L**	0.287	0.339	0.335	0.292	0.058	0.02	0.07	0.63
**BHBA, mmol/L**	0.151	0.212	0.183	0.180	0.552	<0.01	0.88	0.95
***0 h post-feeding, mg/dL***								
**Glucose**	49.2	47.7	48.6	48.4	4.05	0.42	0.35	0.66
**Urea**	33.6	42.3	39.3	36.6	6.04	<0.01	0.27	0.83
***4 h post-feeding, mg/dL***								
**Glucose**	47.1	44.0	47.9	44.2	5.38	0.16	0.09	0.13
**Urea**	35.8	38.5	36.8	37.6	5.10	0.14	0.70	0.55
***Uterine hemodynamics***								
***Measurements at 230 d of gestation (end of TMR supplementation period)***								
**Pulsatility index**	0.69	0.74	0.75	0.68	0.03	0.14	0.05	0.03
**Female**	0.69^b^	0.81^a^						
**Male**	0.70^b^	0.67^b^						
**Resistance index**	0.47	0.49	0.50	0.47	0.01	0.12	<0.01	0.01
**Female**	0.47^b^	0.53^a^						
**Male**	0.47^b^	0.46^b^						
**Systolic/diastolic ratio**	1.94	2.00	2.03	1.92	0.05	0.16	0.03	0.02
**Female**	1.94^b^	2.11^a^						
**Male**	1.95^b^	1.90^b^						
***Measurements at 270 d of gestation (prepartum)***								
**Pulsatility index**	0.57	0.57	0.61	0.53	0.03	0.81	0.05	0.44
**Resistance index**	0.45	0.46	0.47	0.44	0.01	0.45	0.05	0.45
**Systolic/diastolic ratio**	1.99	1.82	1.99	1.82	0.08	0.02	0.03	0.02
**Female**	2.16^a^	1.83^b^						
**Male**	1.82^b^	1.82^b^						

1 Total mixed ration containing grass silage and dried distillers’ grains.

2 Total mixed ration containing grass silage, soybean hulls, and urea.

### Uterine hemodynamics

At 230 d of gestation, FR × OS interactions were detected for the pulsatility index, resistance index, and systolic/diastolic ratio (*P *≤ 0.03; Table 5). In all cases, cows fed SH + urea and carrying female fetuses exhibited greater vascular resistance parameters compared with all other groups. At 270 d of gestation, no FR × OS interactions were observed for pulsatility or resistance indices (*P *≥ 0.44). However, a significant interaction was detected for the systolic/diastolic ratio (*P *= 0.02), wherein cows fed DDGS-based TMR diet and carrying female fetuses presented greater values compared with the other three treatments (Table 5).

### Cow performance, intake, and milk yield and composition during the cow–calf phase

No significant FR × OS interactions were observed for any of the evaluated variables at 60 or 120 d in milk (*P *≥ 0.13; Table 6). At 60 d in milk, maternal FR did not affect BW, BCS, or nutrient intake (DM, CP, and NDF; *P *≥ 0.19) of beef cows. A trend for greater milk yield was observed in cows fed the SH + urea–based TMR compared with those fed the DDGS-based TMR (*P *= 0.07) at mid-gestation, although no significant effects were detected for milk composition (*P *≥ 0.21; Table 6). At 120 d in milk, BW and BCS were similar between cows fed the DDGS-based and SH + urea-based TMRs during mid-gestation (*P *≥ 0.43). Cows fed the SH + urea–based TMR exhibited greater DM and NDF intakes than those fed the DDGS-based TMR (*P *= 0.05), whereas CP intake did not differ as a function of FR (*P *= 0.56) at 120 d in milk. No effects of maternal diet or offspring sex were detected for milk yield or composition at this time point (*P *≥ 0.10; Table 6).

**Table 6.** Effects of dietary treatments during mid-gestation and offspring sex on performance, intake, and milk yield and composition of beef cows during the cow–calf phase

Item	**Feeding regimen** ** (FR)**		**Offspring sex** ** (OS)**		SEM	*P*-value		
	**DDGS** ** (*n *= 17)**	**SH + Urea** ** (*n *= 16)**	**Female** ** (*n *= 17)**	**Male** ** (*n *= 16)**		FR	OS	FR × OS
***60 d in milk***								
**BW, kg**	508.4	509.8	506.3	512.0	6.29	0.86	0.56	0.76
**BCS**	5.06	5.02	4.95	5.13	0.179	0.87	0.48	0.97
***Intake, kg/day***								
**DM**	12.8	12.6	12.8	12.5	0.570	0.87	0.72	0.90
**CP**	1.43	1.43	1.49	1.37	0.120	0.99	0.49	0.64
**NDF**	8.66	8.21	8.48	8.40	0.240	0.19	0.80	0.18
***Milk yield and composition***								
**Milk yield, kg/day**	4.25	5.26	4.57	4.94	0.357	0.07	0.47	0.37
**Protein, %**	3.43	3.40	3.36	3.46	0.073	0.74	0.34	0.68
**Fat, %**	3.08	3.01	3.11	2.99	0.268	0.84	0.76	0.64
**Lactose, %**	4.81	4.80	4.84	4.77	0.100	0.95	0.60	0.21
**Total solids content, %**	12.3	12.1	12.2	12.1	0.335	0.74	0.85	0.56
***120 days in milk***								
**BW, kg**	526.5	528.8	522.0	533.3	5.98	0.77	0.23	0.43
**BCS**	4.96	5.10	4.87	5.19	0.122	0.43	0.07	0.86
***Intake, kg/day***								
**DM**	11.4	12.7	12.3	11.8	0.447	0.05	0.39	0.92
**CP**	1.32	1.40	1.48	1.25	0.095	0.56	0.10	0.92
**NDF**	8.59	9.42	9.19	8.82	0.283	0.05	0.35	0.45
***Milk yield and composition***								
**Milk yield, kg/day**	4.19	4.31	3.92	4.59	0.290	0.76	0.11	0.13
**Protein, %**	3.67	3.54	3.61	3.60	0.067	0.19	0.92	0.80
**Fat, %**	3.95	3.51	3.84	3.62	0.279	0.24	0.59	0.52
**Lactose, %**	4.65	4.75	4.77	4.64	0.076	0.35	0.23	0.52
**Total solids content, %**	13.3	12.8	13.2	12.8	0.333	0.28	0.40	0.73

### Offspring intake, performance, and tissue accretion during cow–calf phase and backgrounding phases

No significant FR × OS interactions were observed for any of the evaluated parameters across the cow–calf or backgrounding phases in the offspring (*P *≥ 0.11; Table 7). During the cow–calf phase, maternal prenatal FR nor offspring sex affected forage intake in the offspring (DM, CP, and NDF; *P *≥ 0.24). Offspring BW at birth, 10, 60, 120, and 210 d of age was not influenced by maternal FR during gestation (*P *≥ 0.27). However, a significant effect of offspring sex was observed from 60 d of age onward, with males showing greater BW than females (*P *≤ 0.09). Likewise, males exhibited greater ADG than females throughout the preweaning period (*P *< 0.001). No FR effects were detected for ultrasound measurements during cow-calf phase (*P *≥ 0.16). During backgrounding, the OS also influenced BW at all feedlot timepoints (35, 65, and 95 d), with males being heavier than females (*P *< 0.001). The total ADG during the backgrounding phase tended to be higher in calves from dams fed DDGS-based TMR diet (*P *= 0.05) and was greater in males than females (*P *= 0.01). No effects of FR, OS, or their interaction were detected for ultrasound measures at the end of the backgrounding phase (*P *≥ 0.11).

**Table 7.** Feed intake, performance, and nutrient accretion of offspring according to maternal nutritional treatment during mid-gestation and offspring sex

Item	**Feeding regimen** ** (FR)**		**Offspring sex** ** (OS)**		SEM	*P*-value		
	**DDGS** ** (*n *= 17)**	**SH + Urea** ** (*n *= 16)**	**Female** ** (*n *= 17)**	**Male** ** (*n *= 16)**		FR	OS	FR × OS
***Cow-calf phase***								
***Forage Intake, kg/day***								
**DM**	4.20	3.81	4.02	3.99	0.270	0.31	0.94	0.31
**CP**	0.49	0.42	0.48	0.44	0.050	0.34	0.57	0.42
**NDF**	3.18	2.85	3.00	3.02	0.232	0.31	0.94	0.24
***Body weight, kg***								
**Birth**	33.9	32.0	31.2	34.7	1.28	0.28	0.06	0.69
**10 d**	43.3	43.9	43.5	43.7	0.925	0.60	0.90	0.88
**60 d**	92.1	95.0	88.3	98.9	2.94	0.47	0.01	0.43
**120 d**	145.1	150.2	139.7	155.5	3.33	0.27	<0.01	0.20
**210 d (Adjusted weaning BW)**	193.8	190.8	179.4	205.3	10.3	0.83	0.09	0.39
***ADG, kg/day***	0.904	0.952	0.837	1.019	0.023	0.14	<0.001	0.15
***Ultrasound scans***								
**Ribeye area, cm²**	30.3	26.8	29.4	27.7	1.83	0.16	0.51	0.77
**Fat thickness in Ribeye area, mm**	0.149	0.159	0.157	0.150	0.015	0.63	0.76	0.52
**Rump muscle length, cm²**	5.64	5.63	5.49	5.79	0.120	0.96	0.08	0.55
**Rump fat thickness, mm**	0.228	0.254	0.253	0.229	0.019	0.28	0.37	0.75
***Backgrounding***								
**Intake, *kg/day***								
**DM**	7.50	5.33	6.48	6.36	0.344	<0.001	0.78	0.93
**CP**	1.57	1.49	1.41	1.65	0.079	0.43	0.03	0.69
**NDF**	5.00	5.28	4.73	5.55	0.266	0.43	0.03	0.67
***Body weight, kg***								
**35 d in the feedlot**	213.1	217.4	193.0	237.4	4.91	0.52	<0.001	0.76
**65 d in the feedlot**	244.5	247.1	220.9	270.8	5.93	0.75	<0.001	0.76
**95 d in the feedlot**	284.5	283.7	254.8	313.4	8.57	0.95	<0.001	0.82
**Total ADG in feedlot, kg**	104.6	91.42	88.9	107.1	4.75	0.05	0.01	0.46
***Ultrasound scans***								
**Ribeye area, cm²**	34.6	32.9	33.8	33.7	2.17	0.56	0.98	0.68
**Fat thickness in Ribeye area, mm**	0.282	0.219	0.279	0.223	0.031	0.11	0.17	0.12
**Rump muscle length, cm²**	7.04	6.83	6.82	7.04	0.240	0.50	0.48	0.11
**Rump fat thickness, mm**	0.465	0.416	0.473	0.408	0.052	0.44	0.36	0.33

## Discussion

The present study provides strong evidence that the choice of feedstuffs used in the formulation of TMR silages markedly influenced the fermentation profile of the preserved forage and, consequently, the physiological responses of pregnant beef cows. Feeding DDGS-based TMR increased voluntary feed intake, likely due to its superior preservation status and improved sensory characteristics (Bandla et al., 2024). This response was consistent with the fermentation profile of DDGS- based TMR silage, which contained greater lactic acid and less butyric acid, indicative of a predominantly homofermentative process (Bandla et al., 2024). Such a profile promotes rapid acidification, inhibits undesirable microrganisms, and preserves soluble nutrients, thereby enhancing greater feed intake (Gerlach et al., 2014; Bernardes et al., 2018; Gusmão et al., 2018; Daniel et al., 2019; Bandla et al., 2024). Moreover, the ensiling of complete diets induces changes in both the composition and the availability of nutrients (Bueno et al., 2020). Thus, silages prepared with soybean hulls plus urea were prone to greater ammonia accumulation due to rapid urea hydrolysis, which increases buffering capacity, delays pH decline, and favors clostridial development during ensiling (Buxton and O’Kiely, 2003; Lazzari et al., 2021). These undesirable conditions intensify proteolysis, elevate NH_3_-N, and reduce the retention of true protein, ultimately impairing nutrient preservation and lowering intake potential.

Beyond intake, cows fed DDGS-based TMR exhibited greater total-tract DM digestibility, underscoring the superior efficiency of nutrient utilization under this feeding strategy. Interestingly, CP digestibility was compromised in cows carrying female fetuses when fed the soybean hull + urea TMR silage, whereas no such effect was observed in cows carrying males. This sex-dependent response suggests that fetal sex may influence maternal adjustments to dietary challenges, reflecting divergent patterns of nutrient allocation between cows gestating male and female fetuses. However, this finding contrasts with previous studies, such as those by Camacho et al. (2014, 2018), which reported that maternal responses to nutritional restriction and refeeding were primarily determined by the dietary treatment, rather than by fetal sex. Similarly, Copping et al. (2020) found no significant effects of fetal sex on maternal nutrient digestibility, suggesting that the influence of fetal sex may be context-dependent, varying according to the diet, the stage of gestation studied, or the degree of nutritional restriction applied in the dam.

A tendency toward greater microbial protein synthesis was observed in cows fed DDGS-based TMR silage. Although greater intake alone could increase substrate availability for microbial growth (Galyean and Tedeschi, 2014), the more favorable balance between rumen-degradable and undegradable protein in DDGS likely optimized nitrogen capture in the rumen. Importantly, within the silo environment, feedstuffs with a greater proportion of true protein (such as DDGS) contribute to better preservation of protein, as their high content of true protein and RUP is less susceptible to proteolysis (Lazzari et al., 2021). Accordingly, two mechanisms may explain the observed tendency: first, DDGS-based TMR silages increased the post-ruminal supply of absorbable amino acids; and second, RUP-derived nitrogen may indirectly contribute to the ruminal nitrogen pool through enhanced urea recycling, as demonstrated in isotopic studies (Batista et al., 2016), ultimately supporting greater microbial protein synthesis.

Consistent with these mechanisms, nitrogen balance responses revealed that cows fed DDGS-based silage consumed more nitrogen and showed a tendency toward greater fecal nitrogen excretion, yet retained more nitrogen overall, reinforcing the superior efficiency of nitrogen utilization under this feeding strategy. The combination of greater intake, improved digestibility, and enhanced nitrogen balance was reflected in distinct physiological consequences for the pregnant beef cows during mid gestation. Those fed the DDGS-based TMR maintained greater performance and BCS during mid-gestation and exhibited a more favorable metabolic profile. Lower circulating concentrations of NEFA and BHBA indicated a reduced reliance on lipid mobilization (Rodriguez et al., 2021; Meneses et al., 2022), suggesting that these cows depended less on body reserves to support metabolic requirements than those fed SH + urea–based TMR diet. In contrast, the greater preprandial plasma urea concentrations observed in cows fed the SH + urea silage are consistent with increased amino acid catabolism, likely of myofibrillar origin, as a compensatory mechanism to supply energy under nutritionally limiting conditions. Such response is in line with the notion that, when dietary protein is insufficient, pregnant cows intensify amino acid deamination to generate carbon skeletons for gluconeogenesis while providing nitrogen for urea synthesis, ultimately reflecting a less efficient use of dietary protein (Reynolds, 1992; Meneses et al., 2022), Nevertheless, the lack of differences in plasma urea concentrations 4 h after feeding suggests that this catabolic response was transient and primarily associated with short-term preprandial insufficiency.

Additionally, a sex-dependent response was observed for maternal circulating IGF-1 and insulin concentrations during mid-gestation. This interaction indicates that maternal endocrine profile is modulated not only by dietary quality but also by fetal sex, reinforcing the concept that male and female fetuses impose distinct adaptation in response to in utero conditions (Trivers and Willard, 1973; Kalisch-Smith et al., 2017; Barcelos et al., 2022; Meneses et al., 2022, 2024; Miranda et al., 2023). Consistent with this, a tendency for greater weights of gestational components was detected in cows carrying male fetuses compared with those carrying females during mid-gestation, supporting the notion of a stronger anabolic drive imposed by male offspring (Thomas et al., 2000; Alur, 2019; Nascimento et al., 2024). Such differences may explain the divergent maternal adaptations in IGF-1 and insulin dynamics. Interestingly, higher IGF-1 concentrations were detected in cows fed DDGS and carrying female fetuses, as well as in cows fed SH + urea and carrying male fetuses, when compared with DDGS-fed cows carrying males. These patterns suggest that maternal IGF-1 may operate as a compensatory mechanism under specific maternal–fetal contexts: in the case of females, to favor their development given their role in herd perpetuation (Rosenfeld and Roberts, 2004; Ithurralde et al., 2019) and in the case of males, to counterbalance the lower availability of dietary substrates from the SH + urea diet. Conversely, other studies in beef cattle did not report differences in maternal metabolite or hormone concentrations based on fetal sex (Copping et al., 2020; Costa et al., 2021; Redifer et al., 2024). These discrepancies could be associated with the degree of nutritional restriction achieved or the duration of treatment, applied, factors that can attenuate or mask differential endocrine responses between fetal sex. In this context, the results of the present study provide valuable evidence by demonstrating that, under specific feeding conditions and gestational stage, fetal sex may play a modulating role on maternal endocrine adaptation, contributing to the understanding of fetal programming mechanisms in cattle.

Despite the benefits in maternal performance observed during mid-gestation, differences in BW between treatments had disappeared by calving. This outcome reflects the fact that all cows were subsequently exposed to the same nutritional management and maintained on high-quality pasture during late gestation, a period in which dietary uniformity tends to override previous differences. Nevertheless, cows that received the DDGS-based TMR during mid-gestation retained an advantage in terms of BCS at parturition, suggesting that they partitioned nutrients preferentially toward maternal tissue reserves rather than to gestational components. Contrary to our initial expectation, this greater maternal condition did not translate into increased fetal growth, as no differences were detected in gestational component weights or calf birth weight. We hypothesized that cows with superior BW and BCS during mid-gestation would mobilize a greater proportion of reserves during late gestation to support conceptus growth, consistent with the principle of homeorhesis (Gionbelli et al., 2024; Moreira et al., 2025). However, in this context of adequate late-gestation nutrition, nutrient mobilization appears to have been minimized, leading to the preservation of maternal reserves instead of enhanced fetal nutrient accretion.

Regarding uterine hemodynamics, during mid-gestation cows carrying female fetuses and fed SH + urea silage exhibited greater resistance, pulsatility, and systolic/diastolic indices, indicating impaired uteroplacental perfusion. This suggests that, under less favorable maternal nutritional conditions, female fetuses are more sensitive to reductions in blood supply, in agreement with the concept that increased uterine and umbilical blood flow and the consequent reduction in vascular resistance are essential indicators of adequate fetal development (Veiga et al., 2018). Conversely, Camacho et al. (2018) and Meneses et al. (2024) reported significant effects of maternal nutritional treatment on uterine and umbilical blood flow; however, no effects of fetal sex were observed in cows subjected to nutritional restriction and refeeding during gestation, indicating that maternal uteroplacental perfusion was more influenced by nutritional status than by fetal sex. In contrast, when all cows were managed under the same adequate nutritional conditions, these differences were attenuated, highlighting the compensatory plasticity of uteroplacental circulation near term. Nevertheless, a residual interaction between fetal sex and maternal diet previously offered during mid-gestation was still observed for the systolic/diastolic ratio, which was greater in cows carrying female fetuses that had received DDGS-based TMR silage. A possible explanation is that, despite the superior nutritional preservation and protein supply provided by DDGS-based TMR silage, female fetuses may exhibit distinct hemodynamic strategies in late gestation, potentially reflecting adaptive adjustments not required by males. Therefore, these responses suggest that maternal diet during mid-gestation exerts transient but sex-dependent effects on uteroplacental hemodynamics, with female fetuses showing heightened vascular sensitivity under both unfavorable and favorable nutritional conditions.

During the cow–calf phase, the lack of FR × OS effects on lactational performance indicates that the sex-dependent adjustments were more restricted to gestation and dissipated after parturition, when placental signals (e.g., placental lactogen and estrogens) cease and the maternal endocrine milieu shifts to the classic homeorhetic state of early lactation (Neville et al., 2001; Schuler et al., 2018). Moreover, the small, early-postpartum tendency for greater milk yield in cows previously fed the SH + urea-based TMR likely reflects short-term compensatory repartitioning after a less favorable intra-gestational environment, rather than a persistent residual effect. Notably, this tendency at 60 DIM was not accompanied by differences in DMI, CP, or NDF intake between feeding regimens, whereas at 120 DIM cows previously assigned to SH + urea based TMR consumed more DM and NDF yet produced similar milk to DDGS cows. Critically, mammary development (alveologenesis and secretory differentiation) accelerates in late gestation (Neville et al., 2001); thus, when all cows received adequate and uniform nutrition near term, any mid-gestation dietary imprint on mammary morphogenesis was largely overridden. This interpretation aligns with evidence that restrictive plans late in gestation depress udder weight, colostrum accumulation, and milk yield (Mellor and Murray, 1985; Mellor et al., 1987; Banchero et al., 2006). By contrast, we are not aware of direct studies isolating mid-gestation effects under adequate late-gestation nutrition in beef cows; thus, based on the present data, mid-gestation challenges appeared to exert at most transient carry-over. Maternal nutrition and body condition can also modulate colostrum/milk composition (total solids, protein, lactose, urea N, somatic cells, and IgG), but such effects are most evident when the restriction extends into late gestation (Swanson et al., 2008; Meyer et al., 2011). Consistent with this framework, prenatal dietary treatments in the present study did not alter milk composition or sustained yield in the subsequent lactation period.

Maternal nutrition during mid-gestation elicited clear physiological and metabolic adaptations in the dam; however, a central question of this study is whether these changes translated into long-term developmental benefits for their offspring. Because mid-gestation coincides with secondary myogenesis and the establishment of critical developmental trajectories (Costa et al., 2021a; Barcelos et al., 2022; Nascimento et al., 2022; Santos et al., 2022; Miranda et al., 2025), it was anticipated that enhanced nutrient supply from DDGS-based TMR silages could positively influence postnatal performance. Yet, the results revealed a more nuanced outcome. Although maternal advantages in physiology, metabolism, and performance were evident during mid-gestation, these responses did not consistently translate into improvements in offspring birth weight, nutrient accretion, growth during the cow–calf phase, or BW during backgrounding. While the present study did not directly investigate molecular mechanisms underlying myogenesis, previous evidence suggests that maternal nutrition during this developmental window can influence myogenic commitment through epigenetic regulation of transcriptional networks controlling the balance between myogenic and fibro-adipogenic lineages (Du et al., 2015; Jennings et al., 2016; Carvalho et al., 2022; Miranda et al., 2023; Nascimento et al., 2024; Gundersen and Anas, 2025; Miranda et al., 2025). Nevertheless, despite the potential for such mechanisms to occur, the anticipated programming effects were not expressed as consistent differences in growth traits. Importantly, during backgrounding, offspring from dams fed DDGS-based TMR during mid-gestation exhibited greater ADG and feed intake, although these responses were not accompanied by differences in BW. Taken together, these findings indicate that different TMR silages offered during mid-gestation can elicit detectable effects on offspring performance, but their ability to promote sustained phenotypic changes appears limited under the conditions evaluated.

## Conclusion

Mid-gestation supplementation with DDGS-based TMR silage increased voluntary feed intake, improved nutrient preservation, and promoted more favorable maternal adaptations compared with soybean hull-based TMR silage. This strategy reduced reliance on maternal tissue reserves, supported better metabolic balance during pregnancy, and helped maintain body condition at calving. The carry-over effects on offspring were modest, suggesting that the nutritional strategies evaluated primarily enhanced maternal efficiency rather than markedly altering postnatal performance. By increasing feed intake, DDGS-based TMR silage not only improved nutrient availability but also supported overall maternal resilience during a critical stage of gestation. Incorporating agro-industrial by-products such as DDGS into TMR silages represents a feasible alternative to mitigate seasonal feed shortages in tropical beef systems, contributing to improved cow–calf productivity and more sustainable management practices.

## Abbreviations:

ADG: average daily gain
BCS: body condition score
BHBA: beta-hydroxybutyrate
BW: body weight
BWp: pregnant body weight
CP: crude protein
DDGS: dried distillers’ grains plus soluble
DIM: days in milk
DM: dry matter
EBW: empty body weight
EBWnp: non-pregnant empty body weight
EBWp: pregnant empty body weight
EE: ether extract
FR: feeding regimen
FR × OS: interaction between prenatal feeding regimen and offspring sex
GUdp: gravid uterus
IDNF/iNDF: indigestible neutral detergent fiber
IGF-1: insulin-like growth factor 1
LMA: longissimus muscle area
MCP: microbial crude protein
NDF: neutral detergent fiber
NEFA: non-esterified fatty acids
NFC: non-fiber carbohydrates
OM: organic matter
OS: effect of offspring sex
PI: pulsatility index
PREG: gestational components
RI: resistance index
RML: rump muscle length
S/D: systolic/diastolic ratio
SFT: subcutaneous fat thickness
SH: soybean hulls
TDN: total digestible nutrients
TiO_2_: titanium dioxide
TMR: total mixed ration
